# Big datasets of optical-wireless cyber-physical systems for optimizing manufacturing services in the internet of things-enabled industry 4.0

**DOI:** 10.1016/j.dib.2022.108026

**Published:** 2022-03-09

**Authors:** Muhammad Faheem, Rizwan Aslam Butt

**Affiliations:** a Department of Computer Engineering, Abdullah Gul University, Kayseri, 38080, Turkey; b Department of Electronics Engineering, NED University, Karachi 75270, Pakistan

**Keywords:** Internet of things, Big data, Optical sensor network, Wireless sensor network, Industry 4.0

## Abstract

The Industry 4.0 revolution is aimed to optimize the product design according to the customers' demand, quality requirements and economic feasibility. Industry 4.0 employs advanced two-way communication technologies for optimizing the manufacturing process to increase the sales of the products and revenues to cope the existing global economy issues. In Industry 4.0, big data obtained from the Internet of Things (IoT)-enabled industrial Cyber-Physical Systems (CPS) plays an important role in enhancing the system service performance to boost the productivity with enhanced quality of customer experience. This paper presents the big datasets obtained from the Internet of things (IoT)-enabled Optical-Wireless Sensor Networks (OWSNs) for optimizing service systems' performance in the electronics manufacturing Industry 4.0. The updated raw and analyzed big datasets of our published work [3] contain five values namely, data delivery, latency, congestion, throughput, and packet error rate in OWSNs. The obtained dataset are useful for optimizing the service system performance in the electronics manufacturing Industry 4.0.

## Specifications Table

**Table:** 

Subject	Computer Science: Computer Networks and Communications
Specific subject area	Optical-wireless communication in the electronics manufacturing Industry 4.0.
Type of data	Graphs and Tables
How the data were acquired	Data was captured using Internet of things-enabled optical-wireless sensor networks in the electronics manufacturing Industry 4.0.
Data format	Raw and analyzed optical-wireless sensors data in an electronics manufacturing Industry 4.0.
Description of data collection	The big data sets were collected by optical-wireless sensor networks deployed on different types of manufacturing and assembly systems in the electronics Industry 4.0. To collect the big data in a particular scenario, a static topology by taking into account the line-of-sight and the non-line-of-sight issues was considered in an indoor industrial environment. To gather real-time big data from the systems involved in the electronics manufacturing process a cobot, i.e., the static sink was deployed in a specific location in the plant. The remote user can access and configure both wireless and optical nodes by connecting to the cobot through the intranet or the internet communication technologies such as the 5G. Distinct from the existing sink, the cobot can intelligently monitor, learn and configure the entire deployed network by closely monitoring the human interventions. Thus, the cobot minimizes the user interventions in the whole big data gathering process in Industry 4.0.
Parameters for data collection	The data was gathered in day and night by employing wireless and optical sensors numbering 450 and 100, respectively. The wireless sensor nodes are equipped with physical layer standard IEEE 802.15.4 and frequency 2.4 GHz unlicensed industrial, scientific and medical (ISM) band. The optical nodes are equipped with physical layer standard IEEE 802.15.7 using light wavelengths from 7000 nm to 300 nm (LED technology), which varies based on the applications. In addition, the group leader nodes are equipped with both physical layer standards IEEE 802.15.4 and IEEE 802.15.7 for wireless and optical communication in the network.
Data source location	City/Town/Region: Kayseri/Kocasinan, Country: Turkey, Latitude and longitude (and GPS coordinates, if possible) for collected samples/data: N38 °71′ and E35 °43′.
Data accessibility	Data repository name: Mendeley Data identification number: DOI:10.17632/8kvdbhrgxt.3 Direct URL t to data: https://data.mendeley.com/datasets/8kvdbhrgxt/3
Related research paper	M. Faheem, R. A. Butt, R. Ali, B. Raza, M. A. Ngadi, and V. C. Gungor, ``CBI4. 0: A Cross-layer Approach for Big Data Gathering for Active Monitoring and Maintenance in the Manufacturing Industry 4.0,'' *Journal of Industrial Information Integration,* p. 100236, 2021. https://doi.org/10.1016/j.jii.2021.100236

## Value of the Data

• The data presented in the article provides a fundamental building block of the next-generation Internet of things-enabled optical-wireless communication architectures for big data gathering in the electronics manufacturing Industry 4.0.
• The published data will guide scientists for low-cost and energy efficient integration of different types of cyber-physical systems with varying data capacity requirements, and operate them optimally within realistic network scenarios in the electronics manufacturing Industry 4.0.
• The data presented in the article will serve as a guide for readers for closely monitoring the assembly and manufacturing processes in real-time to minimize the faulty products and to boost the production process with lesser human interventions in the electronics manufacturing Industry 4.0.
• The published data can be used as a benchmark problem by researchers interested in artificial intelligence-based network analysis of different types of manufacturing systems in the manufacturing Industry 4.0.

## 1 Data Description

Internet of things is an emerging domain that promises ubiquitous connection of various devices to the Internet in several industrial applications like e-health, manufacturing, logistics, and utilities [1], [2], [3]. However, the accuracy of obtaining the big data from the IoT-enabled OWSNs is very challenging due to moving objects, obstacles, line-of-sight, and non-line-of-sight issues in an electronics manufacturing Industry 4.0 [4], [5], [6], [7], [8]. The offered dataset in this article provides essential information for real-time observations of the electronics manufacturing process in an electronics manufacturing Industry 4.0. The offered datasets guide the researchers about how to identify the faulty systems placed in various positions. Thus, it allows the system monitoring and control personnel to take appropriate actions for improving the quality and quantity of the product to meet customer demands. The data offered in this article were collected using wireless and optical sensors placed in different positions on different electronics manufacturing and assembly systems in an indoor industrial environment. In the deployed network, each node is responsible to observe the surroundings and collaborates with the neighboring node to forward the sensed information to the cobot. Unlike the traditional sink, the cobot is an intelligent device that can learn from human actions and perform actions on demand. Therefore, the deployed optical-sensor network requires less human intervention in the monitoring and control processes.

**Fig. 1:**
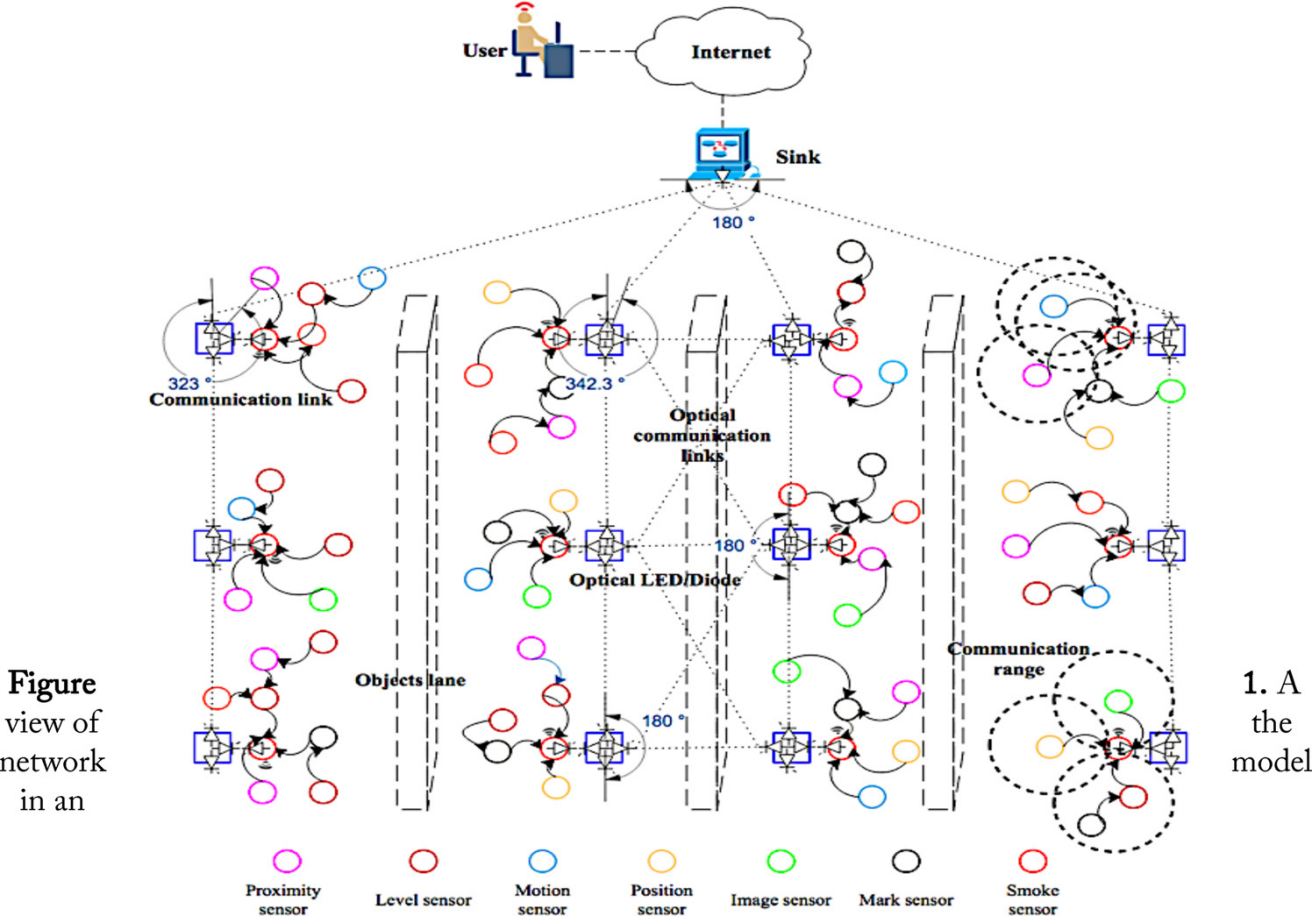
A view of the network model in an electronics manufacturing Industry 4.0 [3].

Fig. 1 describes the network model deployed in Industry 4.0. In Fig. 1, the colored circle shape icons indicate the different types of sensor nodes, e.g., proximity sensor, level sensor, motion sensor, position sensor, etc. In particular, the red-colored circle icon is equipped with both wireless and optical line of sight characteristics compared to the reset of sensors, which only can communicate wirelessly in the network. The dotted circle shows the communication range of a sensor node embedded in the manufacturing systems for fault monitoring purposes. The blue-colored box icons show optical sensors equipped with multiple led in different lines of directions. The solid arrows and light black color dotted lines show the wireless and optical communication, respectively. The computer-like icon is a cobot (sink), which is equipped with optical sensors to communicate with the rest of the deployed network. The cloud-like icon indicates the Internet with different types of networks. Consequently, the cobot is equipped with 5G communication technology to communicate with the Internet. Consequently, a remote user using Internet of Services (IoS) and IoT such as 5G bi-directional communication links can interact with the deployed network to directly configure, monitor, control, and configure the network.

**Fig. 2:**
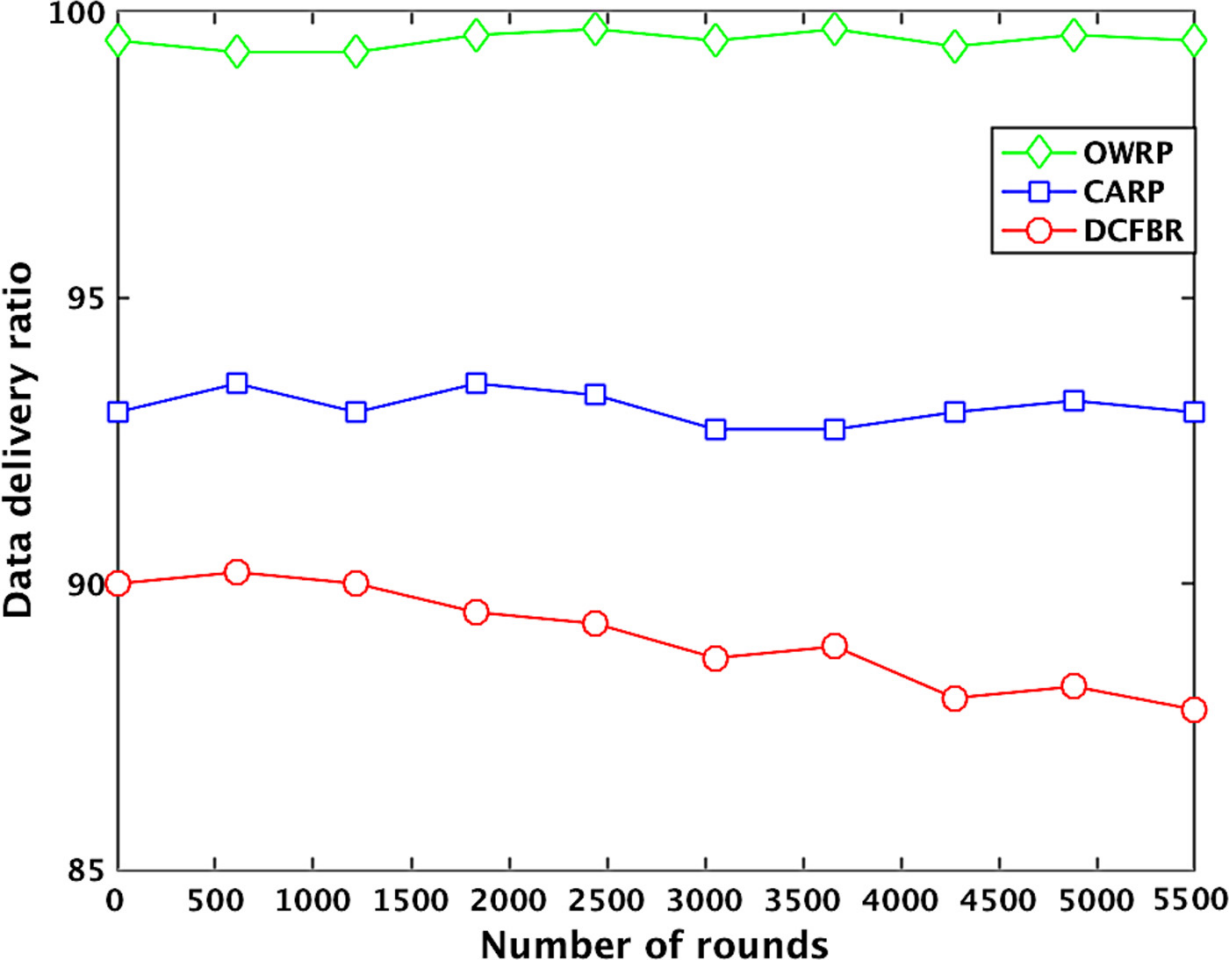
Effect of number of rounds to data delivery

Table 1 describes the datasets related to the ratio of data delivery in OWSNs. It clearly shows that the data delivery ratio (DDR) of OWRP in the initial rounds between 1 and 1000 is high around 99.95% compared to 93.15% in CARP. However, the DDR value of OWRP is decreasing from 99.15%, 99.81%, 99.83%, 99.59 %, and to 99.25% when the round numbers are between 2000 and 5000 in the network. However, the datasets show that the value of DDR is decreasing rapidly from 93.14%, 93.46%, 92.73%, 93.06%, and to 91.68% in CARP compared to the OWRP scheme in the network. On the other hand, the DDR value of DCFBR is reducing up to 90.93%, 88.83%, 87.26%, 85.50%, 85.69%, and 84.60% between round numbers 100 and 1000, 1001 and 2000, 2001 and 3000, 3001 and 4000, 4001 and 5000, and 5001 and 5500, respectively, in the network. The average of obtained PDR big datasets graphically is shown in Fig. 2.

**Fig. 3:**
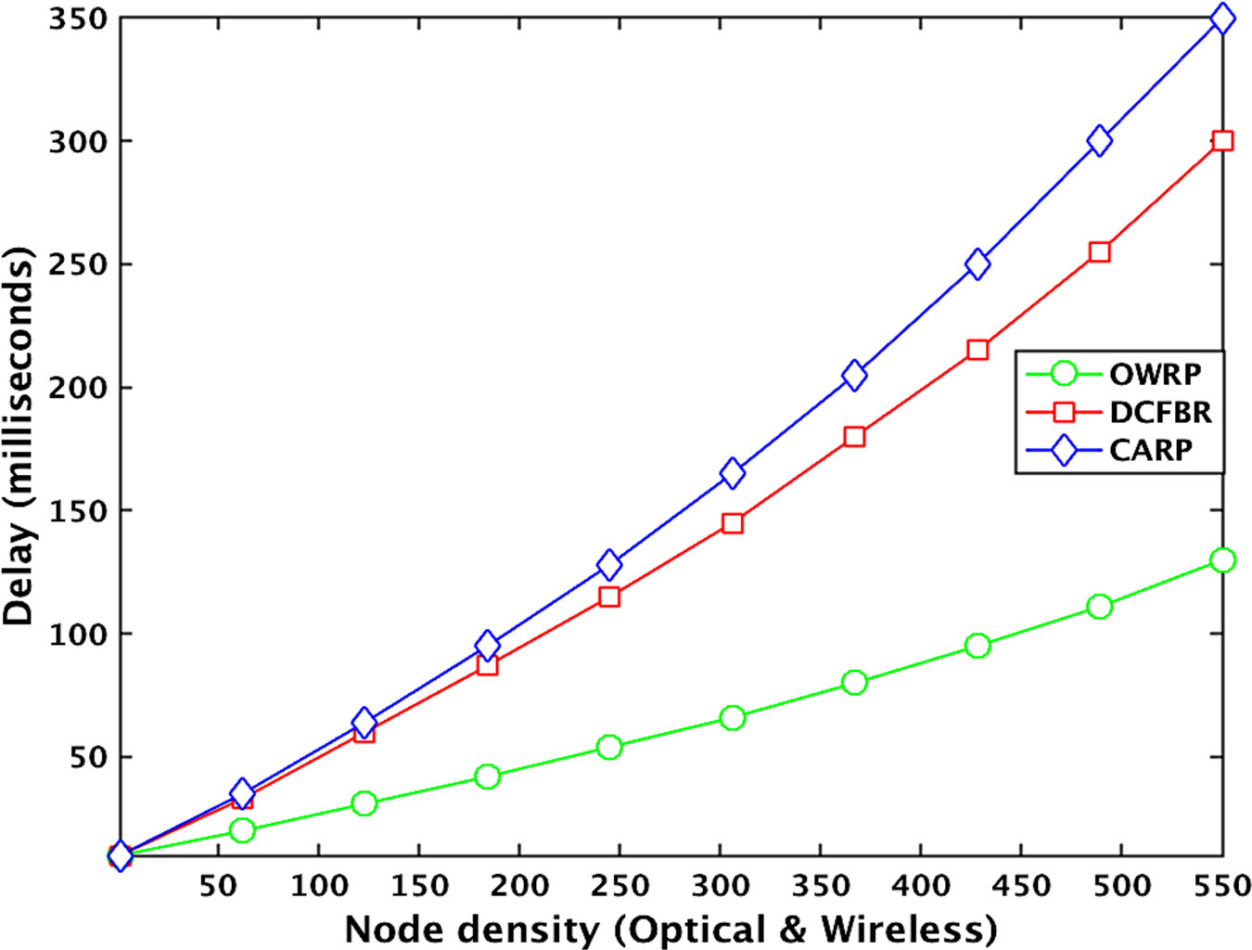
Effect of node density to network delay

Table 2 describes the datasets related to the latency in the OWSNs. The obtained big datasets illustrate that the latency value (LV) of OWRP with node density between 1 and 100 is low around 30ms compared to 63ms in CARP. However, the latency value of OWRP is increasing around 48ms, 66ms, 85ms, 117ms, and 131ms when the numbers optical-wireless sensor nodes are between 110 and 550 in the network. The datasets show that the LV is increasing rapidly around 63ms, 98ms, 170ms, 235ms, 291ms, and 350ms in CARP compared to the OWRP scheme in the network. On the other hand, the LV of DCFBR is noticed around 62ms, 89ms, 137ms, 186, 267ms, 308ms with number of nodes between 10 and 100, 101 and 200, 201 and 300, 301 and 400, 401 and 500, and 501 and 550, respectively, in the network. The average of obtained LV big datasets graphically is shown in Fig. 3.

**Table 1:** Datasets for packet delivery ratio in OWSNs.

No. of rounds	Packet delivery ratio values					
Protocols	OWRP	Avg. ≅ (%)	CARP	Avg. ≅ (%)	DCFBR	Avg. ≅ (%)
100	0.009995		0. 009315		0. 009028	
200	0.009989		0. 009387		0. 009276	
300	0. 009989		0. 009344		0. 009295	
400	0. 009988		0. 009369		0. 009168	
500	0. 009966	0. 009978	0. 009361	0. 009361	0. 009068	0. 009093
600	0. 009990		0. 009282		0. 009077	
700	0. 009959		0. 009477		0. 009133	
800	0. 009997		0. 009206		0. 009074	
900	0. 009959		0. 009397		0. 008906	
**1000**	**0.** 00**9948**		**0.** 00**9472**		**0.** 00**8903**	
1100	0. 009969		0. 008968		0. 008916	
1200	0. 009897		0. 009096		0. 008995	
1300	0. 009992		0. 009292		0. 008915	
1400	0. 009950		0. 009248		0. 008994	
1500	0. 009880	0. 009962	0. 009429	0. 009315	0. 008849	0. 008883
1600	0. 009998		0. 009365		0. 008878	
1700	0. 009973		0. 009467		0. 008837	
1800	0. 009997		0. 009191		0. 008893	
1900	0. 009978		0. 009570		0. 008776	
**2000**	**0.** 00**9987**		**0.** 00**9519**		**0.** 00**8776**	
2100	0. 009988		0. 009490		0. 008741	
2200	0. 009991		0. 009247		0. 008774	
2300	0. 009993		0. 009389		0. 008779	
2400	0. 009993		0. 009358		0. 008797	
2500	0. 009983	0. 009981	0. 009334	0. 009346	0. 008696	0. 008726
2600	0. 009998		0. 009487		0. 008781	
2700	0. 009997		0. 009406		0. 008702	
2800	0. 009981		0. 009310		0. 008697	
2900	0. 009981		0. 009115		0. 008616	
**3000**	**0.** 00**9995**		**0.** 00**9325**		**0.** 00**8674**	
3100	0. 009989		0. 009372		0. 008679	
3200	0. 009989		0. 009399		0. 008699	
3300	0. 009979		0. 009299		0. 008694	
3400	0. 009987		0. 008979		0. 008685	
3500	0. 009988	0. 009983	0. 009561	0. 009273	0. 008646	0. 008650
3600	0. 009983		0. 009490		0. 008699	
3700	0. 009986		0. 009151		0. 008559	
3800	0. 009978		0. 009349		0. 008548	
3900	0. 009977		0. 008918		0. 008614	
**4000**	**0.** 00**9975**		**0.** 00**9218**		**0.** 00**8679**	
4100	0. 009995		0. 009378		0. 008679	
4200	0. 009946		0. 009495		0. 008659	
4300	0. 009988		0. 009334		0. 008554	
4400	0. 009989		0. 009283		0. 008582	
4500	0. 009978	0. 009959	0. 009290	0. 009306	0. 008550	0. 008569
4600	0. 009916		0. 008912		0. 008552	
4700	0. 009962		0. 009398		0. 008592	
4800	0. 009957		0. 009268		0. 008537	
4900	0. 009942		0. 009314		0. 008515	
**5000**	**0.** 00**9921**		**0.** 00**9397**		**0.** 00**8464**	
5100	0. 009955		0. 009203		0. 008493	
5200	0. 009912		0. 009282		0. 008472	
5300	0. 009933	0. 009925	0. 009091	0. 009168	0. 008470	0. 008460
5400	0. 009901		0. 009335		0. 008433	
**5500**	**0.** 00**9925**		**0.** 00**8930**		**0.** 00**8430**

**Fig. 4:**
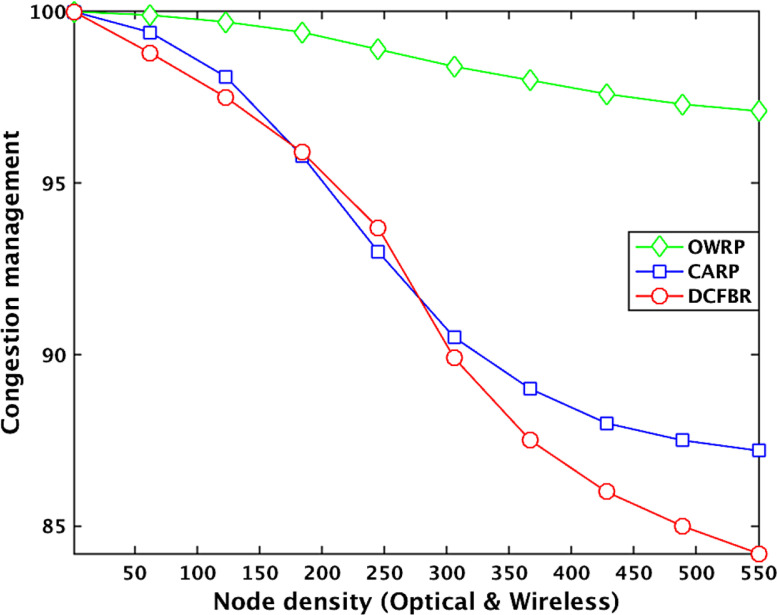
Effect of nodes density on congestion management

Table 3 shows the datasets related to congestion management in the OWSNs. The obtained big datasets illustrate that the congestion management value (CM) of OWRP with node density between 1 and 100 is high around 99.8% compared to 98.8% in CARP. However, the CM value of OWRP is decreasing around 99.5%, 98.6%, 98.7%, 97.4 %, and 97.1% when the numbers optical-wireless sensor nodes are between 110 and 550 in the network. On the other hand, the datasets show that the CM is decreasing rapidly around 96.2%, 91.2%, 87.5%, 86%, and 85.6% in CARP compared to the OWRP scheme in the network. On the other hand, the CM value of DCFBR is recorded around 98.3%, 95.6%, 92%, 86%, 82.3%, and 81.3% with nodes density between 1 and 550 in the network. The average of obtained CM big datasets graphically is shown in Fig. 4.

**Fig. 5:**
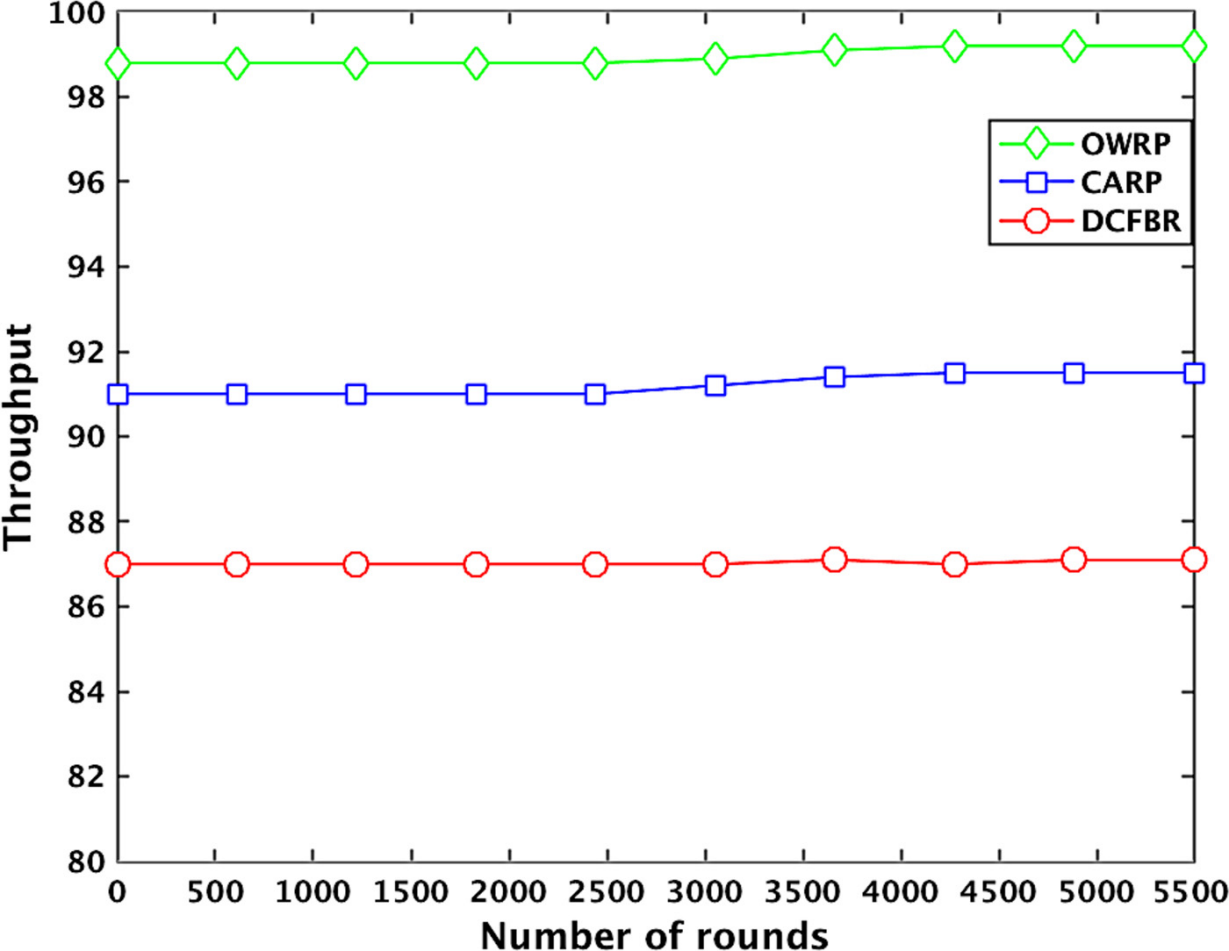
Effect of number of rounds to throughput

Table 4 shows the datasets related to throughput in the OWSNs. The obtained big datasets show that the throughput value (TP) of OWRP with node density between 1 and 100 is high around 99.2% compared to 91.2% in CARP. However, the TP value of OWRP is changing around 99.1%, 98.9%, 98.95%, 98.84 %, and 99.04% when the numbers optical-wireless sensor nodes are between 110 and 550 in the network. The big datasets show that the TP is decreasing rapidly around 91.4%, 90.3%, 90.3% and rising up to 91.7%, and 91.8% in the same round numbers in CARP compared to the OWRP scheme in the network. On the other hand, the TP value in DCFBR is noticed low around 87.8%, 87.5%, 87.4%, 87.4%, 87.1%, and 87.5% between round numbers 100 and 1000, 1001 and 2000, 2001 and 3000, 3001 and 4000, 4001 and 5000, and 5001 and 5500, respectively. The average of obtained TP big datasets graphically is shown in Fig. 5.

**Fig. 6:**
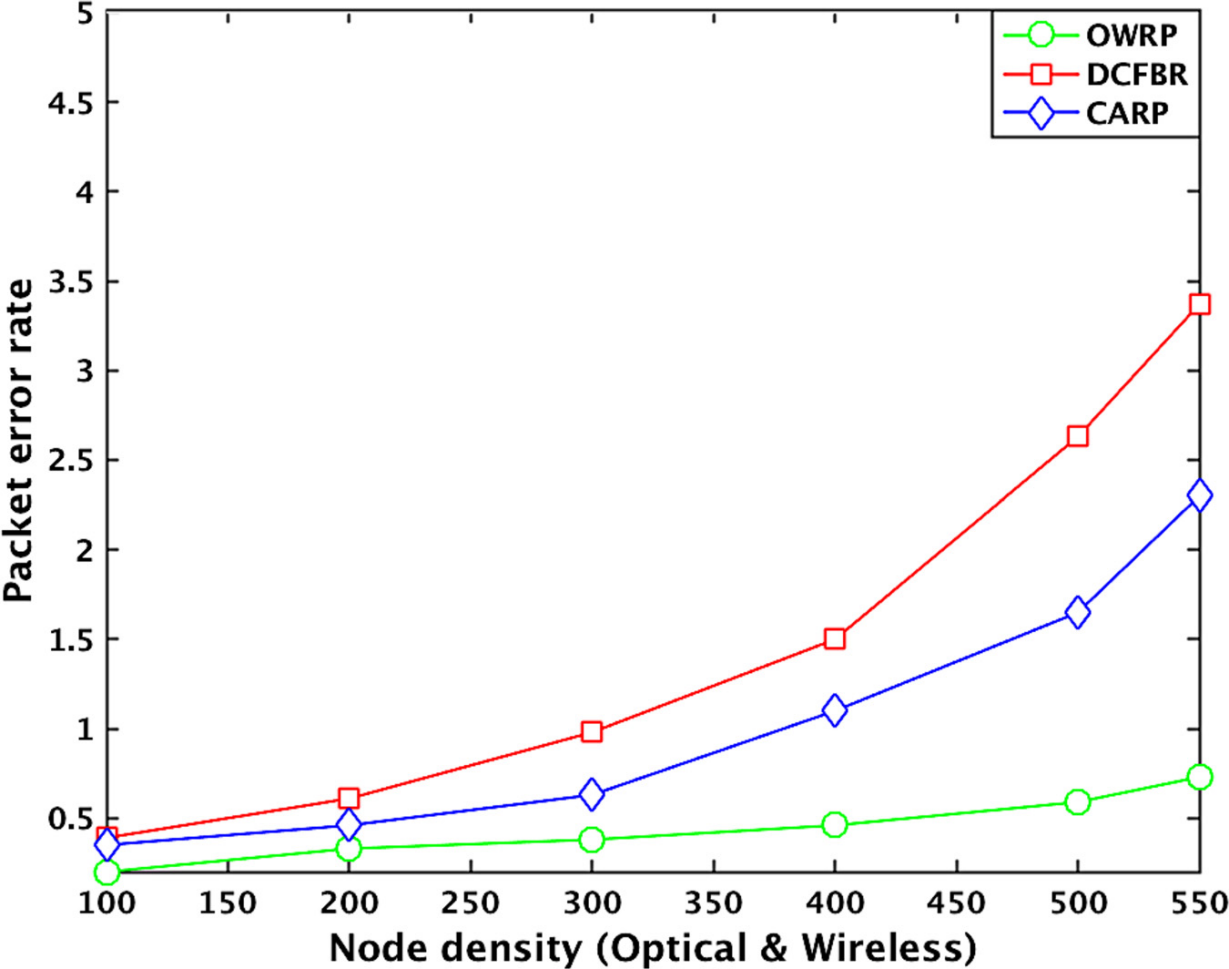
Effect of number of nodes to packet error rate

Table 5 shows the datasets related to packet error rate in the OWSNs. The obtained big datasets show that the packet error rate value (PER) of OWRP with node density between 1 and 100 is low around 0.2% compared to 0.35% in CARP and 0.39% in DCFBR. The PER value of OWRP is changing around 0.33%, 0.38%, 0.46%, 0.59%, and 0.73% when the numbers optical-wireless sensor nodes are between 110 and 550 in the network. Similarly, the PER value of CARP is changing around 0.46%, 0.63%, 1.1%, 1.65%, and 2.3% when the numbers optical-wireless sensor nodes are between 110 and 550 in the network. Compared to all other schemes, the PER value of DCFBR is observed high around 0.61%, 0.98%, 1.5%, 2.63%, and 3.37% between 110 and 550 against the OWRP and CARP in the network. The average of obtained PER big datasets graphically is shown in Fig. 6.

**Table 2:** Datasets for latency in OWSNs.

No. of nodes	Latency values					
Protocols	OWRP	Avg. ≅(ms)	CARP	Avg. ≅(ms)	DCFBR	Avg. ≅(ms)
10	0.001588		0.003957		0.003950	
20	0. 001818		0. 004988		0. 004875	
30	0. 002239		0. 005737		0. 005633	
40	0. 002545		0. 006152		0. 006055	
50	0. 002741	0.002955	0. 006512	0.006312	0. 006310	0.006160
60	0. 003209		0. 006783		0. 006585	
70	0. 003483		0. 007119		0. 006911	
80	0. 003767		0. 007390		0. 007050	
90	0. 004072		0. 007408		0. 007098	
**100**	**0.** 00**4084**		**0.** 00**7519**		**0.** 00**7211**	
110	0. 004289		0. 007680		0. 007320	
120	0. 004356		0. 008290		0. 008001	
130	0. 004467		0. 008850		0. 008222	
140	0. 004483		0. 009190		0. 008560	
150	0. 004593	0.004770	0. 009530	0.009844	0. 008840	0.008942
160	0. 004677		0. 009910		0. 009315	
170	0. 004868		0.010190		0.009695	
180	0. 005099		0. 011020		0. 009723	
190	0. 005378		0. 011770		0. 009772	
**200**	**0.** 00**5489**		0. 0**12010**		0. 00**9964**	
210	0. 005541		0. 012991		0. 010888	
220	0. 005688		0. 014122		0. 010278	
230	0. 005954		0. 015711		0. 012556	
240	0. 006373		0. 016802		0. 012915	
250	0. 006555	0.006582	0. 017677	0.016984	0. 013147	0.013673
260	0. 006792		0. 017934		0. 014155	
270	0. 006879		0. 018381		0. 015394	
280	0. 007169		0. 018593		0. 015587	
290	0. 007378		0. 018788		0. 015872	
**300**	**0.** 00**7489**		0. 0**18842**		0. 0**15941**	
310	0. 007546		0. 018990		0. 015800	
320	0. 007758		0. 019820		0. 016327	
330	0. 008169		0. 020719		0. 016729	
340	0. 008273		0. 021508		0. 016908	
350	0. 008451	0.008544	0. 023077	0.023486	0. 017071	0.018627
360	0. 008672		0. 025137		0. 018188	
370	0. 008778		0. 026085		0. 019088	
380	0. 009135		0. 026393		0. 020399	
390	0. 009266		0. 026588		0. 022522	
**400**	**0.** 00**9393**		0. 0**26547**		0. 0**23240**	
410	0. 011197		0. 026899		0. 023890	
420	0. 011389		0. 027488		0. 024422	
430	0. 011592		0. 028076		0. 025079	
440	0. 010777		0. 028558		0. 025889	
450	0. 010911	0.011703	0. 028975	0.029048	0. 026972	0.026696
460	0. 011975		0. 029483		0. 026480	
470	0. 012078		0. 029689		0. 027688	
480	0. 012138		0. 029798		0. 027703	
490	0. 012389		0. 029968		0. 028900	
**500**	0. 0**12584**		0. 0**31549**		0. 0**29940**	
510	0. 012611		0. 032011		0. 030011	
520	0. 012757		0.033922		0.030901	
530	0. 013135	0.013064	0. 035510	0.035049	0. 030980	0.030782
540	0. 013313		0. 036301		0. 031001	
**550**	0. 0**13501**		0. 037501		0. 031015

**Table 3:** Datasets for congestion management in OWSNs.

No. of nodes	Congestion management values					
Protocols	OWRP	Avg. ≅(%)	CARP	Avg. ≅(%)	DCFBR	Avg. ≅(%)
10	0.009999		0.009999		0.009901	
20	0.009999		0.009970		0.009900	
30	0.009998		0.009961		0.009903	
40	0.009997		0.009852		0.009800	
50	0.009897	0.009975	0.009850	0.009877	0.009840	0.009834
60	0.009895		0.009840		0.009833	
70	0.009993		0.009833		0.009832	
80	0.009990		0.009830		0.009812	
90	0.009989		0.009820		0.009805	
**100**	0.0**09989**		0.0**09815**		0.0**09709**	
110	0.009986		0.009780		0.009700	
120	0.009979		0.009755		0.009702	
130	0.009978		0.009701		0.009661	
140	0.009973		0.009670		0.009630	
150	0.009872	0.009946	0.009653	0.009617	0.009603	0.009560
160	0.009865		0.009620		0.009570	
170	0.009959		0.009569		0.009529	
180	0.009955		0.009546		0.009500	
190	0.009949		0.009470		0.009401	
**200**	0.0**09948**		0.0**09405**		0.0**09304**	
210	0.009941		0.009360		0.009302	
220	0.009915		0.009305		0.009301	
230	0.009880		0.009260		0.009290	
240	0.009865		0.009210		0.009280	
250	0.009848	0.009860	0.009203	0.009115	0.009263	0.009194
260	0.009848		0.009101		0.009251	
270	0.009843		0.009000		0.009190	
280	0.009841		0.008947		0.009021	
290	0.009841		0.008915		0.009082	
**300**	0.0**09841**		0.0**08850**		0.0**08955**	
310	0.009830		0.008844		0.008944	
320	0.009915		0.008820		0.008828	
330	0.009812		0.008812		0.008755	
340	0.009807		0.008801		0.008700	
350	0.009803	0.009866	0.008770	0.008750	0.008630	0.008590
360	0.009801		0.008755		0.008511	
370	0.009795		0.008709		0.008480	
380	0.009791		0.008677		0.008380	
390	0.009791		0.008656		0.008366	
**400**	0.0**09790**		0.0**08651**		0.0**08301**	
410	0.009760		0.008644		0.008300	
420	0.009751		0.008630		0.008288	
430	0.009744		0.008623		0.008253	
440	0.009743		0.008611		0.008251	
450	0.009743	0.009741	0.008601	0.008605	0.008241	0.008225
460	0.009741		0.008600		0.008200	
470	0.009738		0.008589		0.008199	
480	0.009733		0.008587		0.008187	
490	0.009730		0.008581		0.008181	
**500**	0.0**09730**		0.0**08581**		0.0**08150**	
510	0.009728		0.008570		0.008140	
520	0.009722		0.008566		0.008136	
530	0.009719	0.009719	0.008549	0.008555	0.008129	0.008129
540	0.009718		0.008545		0.008125	
**550**	0.009710		0.008544		0.008114

**Table 4:** Datasets for throughput in OWSNs.

No. of rounds	Throughput values					
Protocols	OWRP	Avg. ≅(%)	CARP	Avg. ≅(%)	DCFBR	Avg. ≅(%)
100	0.009891		0.009189		0.008790	
200	0.009875		0.009174		0.008787	
300	0.009888		0.009166		0.008771	
400	0.009871		0.009157		0.008772	
500	0.009899	0.009918	0.009150	0.009150	0.008760	0.008768
600	0.009885		0.009148		0.008772	
700	0.009990		0.009137		0.008760	
800	0.009992		0.009133		0.008762	
900	0.009991		0.009128		0.008750	
**1000**	0.0**09900**		0.0**09119**		0.0**08754**	
1100	0.009901		0.009165		0.008760	
1200	0.009989		0.009146		0.008765	
1300	0.009968		0.009161		0.008722	
1400	0.009966		0.009150		0.008745	
1500	0.009889	0.009908	0.009140	0.009140	0.008767	0.008753
1600	0.009874		0.009158		0.008787	
1700	0.009879		0.009137		0.008734	
1800	0.009801		0.009116		0.008789	
1900	0.009911		0.009120		0.008712	
**2000**	0.0**09898**		0.0**09112**		0.0**08745**	
2100	0.009846		0.009062		0.008761	
2200	0.009867		0.009035		0.008734	
2300	0.009887		0.009014		0.008734	
2400	0.009895		0.009036		0.008745	
2500	0.009888	0.009887	0.009013	0.009026	0.008765	0.008742
2600	0.009998		0.009011		0.008777	
2700	0.009848		0.009002		0.008701	
2800	0.009878		0.009018		0.008741	
2900	0.009876		0.009019		0.008711	
**3000**	0.0**09889**		0.0**09050**		0.0**08753**	
3100	0.009870		0.009045		0.008722	
3200	0.009910		0.009022		0.008724	
3300	0.009911		0.009012		0.008756	
3400	0.009924		0.009023		0.008767	
3500	0.009803	0.009895	0.009045	0.009033	0.008776	0.008744
3600	0.009889		0.009005		0.008737	
3700	0.009899		0.009069		0.008723	
3800	0.009891		0.009022		0.008727	
3900	0.009931		0.009056		0.008754	
**4000**	0.0**09920**		0.0**09031**		0.0**08754**	
4100	0.009869		0.009181		0.008702	
4200	0.009855		0.009156		0.008711	
4300	0.009876		0.009111		0.008722	
44000	0.009940		0.009180		0.008710	
4500	0.009801	0.009884	0.009165	0.009168	0.008701	0.008712
4600	0.009841		0.009178		0.008702	
4700	0.009901		0.009145		0.008701	
4800	0.009977		0.009189		0.008711	
4900	0.009887		0.009187		0.008743	
**5000**	0.0**09888**		0.0**09188**		0.0**08715**	
5100	0.009878		0.009178		0.008765	
5200	0.009920		0.009186		0.008737	
5300	0.009911	0.009904	0.009197	0.009179	0.008726	0.008750
5400	0.00990		0.009165		0.008743	
**5500**	0.009911		0.009170		0.008781

**Table 5:** Datasets for packet error rate in OWSNs.

No. of nodes	Packet error rate values					
Protocols	OWRP	Avg. ≅(%)	CARP	Avg. ≅(%)	DCFBR	Avg. ≅(%)
10	0.001100		0.001498		0.001992	
20	0.001200		0.002588		0.003383	
30	0.001350		0.003694		0.003966	
40	0.001600		0.003789		0.004089	
50	0.001700	0.001986	0.003894	0.003538	0.004124	0.003893
60	0.001900		0.003881		0.004255	
70	0.002100		0.003987		0.004275	
80	0.002600		0.003977		0.004289	
90	0.003110		0.003981		0.004276	
**100**	0.003200		0.004091		0.004283	
110	0.003208		0.004223		0.004356	
120	0.003251		0.004243		0.004754	
130	0.003285		0.004345		0.005187	
140	0.003291		0.004456		0.005579	
150	0.003301	0.003306	0.004534	0.004604	0.006155	0.006145
160	0.003310		0.00459		0.006584	
170	0.003312		0.004765		0.006745	
180	0.003330		0.004878		0.006911	
190	0.003380		0.004989		0.007391	
**200**	0.003393		0.005012		0.007789	
210	0.003458		0.005176		0.007886	
220	0.003531		0.005287		0.008179	
230	0.003616		0.005574		0.008278	
240	0.003688		0.005867		0.008510	
250	0.003756	0.003765	0.006278	0.006346	0.008983	0.009757
260	0.003790		0.006549		0.009491	
270	0.003852		0.006769		0.008782	
280	0.003859		0.006922		0.009979	
290	0.003970		0.007323		0.012710	
**300**	0.004127		0.007711		0.014768	
310	0.004278		0.008067		0.014968	
320	0.004331		0.008567		0.015124	
330	0.004366		0.009078		0.015663	
340	0.004488		0.009387		0.015967	
350	0.004536	0.004629	0.009789	0.010923	0.016276	0.015060
360	0.004620		0.011265		0.016837	
3700	0.004752		0.011456		0.0017223	
380	0.004887		0.012487		0.017627	
390	0.004946		0.013543		0.017954	
**400**	0.005089		0.015634		0.018454	
410	0.005182		0.011543		0.021479	
420	0.005275		0.011932		0.022686	
430	0.005388		0.012430		0.023588	
440	0.005566		0.013511		0.024990	
450	0.005725	0.005886	0.015600	0.016519	0.025691	0.026294
460	0.005908		0.017201		0.026685	
470	0.006160		0.018456		0.027790	
480	0.006367		0.019409		0.028703	
490	0.006518		0.022510		0.029711	
**500**	0.006767		0.022601		0.030612	
510	0.006845		0.022704		0.031619	
520	0.007056		0.022802		0.032830	
530	0.007376	0.007311	0.023311	0.023172	0.033528	0.033742
540	0.007587		0.023751		0.034845	
**550**	0.007689		0.023291		0.035887

## 2 Experimental Design, Materials and Methods

In this work, a set of optical and wireless sensor nodes were statically embedded in different systems located in an area of 285 (length)  ×  110 (width) in the indoor electronics manufacturing industrial environment. The number of optical sensor nodes, compliant to IEEE 802.15.7 physical layer standard and operating on the wavelength from 7000nm to 300nm are set to 100. On the other hand, the wireless sensor nodes, compliant to physical layer standard IEEE 802.15.4 are set to 450. In the deployment, the nodes equipped with both wireless and optical communication technologies act like gateway head nodes and are responsible for gathering observed data from neighboring nodes and forward it to the cobot via optical communication technology. The energy of each wireless node is set to 15J with a communication range of up to 3 to 5m and data rates up to 256 kbps [9]. While the communication range of the optical sensors was set to 10m and data rates up to 1 Gbps. The data packet size of the wireless sensor nodes is set to 72 bytes and uses the Quadrature phase-shift keying (QPSK) modulation mechanism in the network [10]. The memory size of wireless and optical sensor nodes was set to 5Mb and 10Mb, respectively. In addition, the channel and energy consumption model used in this study is the same as discussed in [3,11]. The widely used parameters and values used in existing studies are given in Table 6.

**Table 6:** Simulation parameters and values

Simulation Model Parameters	Values
Simulation tool	EstiNet 12 & MongoDB
Cobot (sink)	1
Wireless sensors	450
Optical sensors	100
Physical layer wireless standard	802.15.4
Physical layer optical standard	802.15.7
Wavelength for optical standard	7000nm to 300nm
Initial sensor node energy	15J
High transmission power	0.46W
Low transmission power	0.31W
Packet receiving power	0.05W
Idle listening	0.023W
Sleeping power	$3\;\times \;10^{-6}$W
Data aggregation	0.019W
Packet length	72 bytes
Wireless data transfer rate	256 kbps
Optical data transfer rate	1Gbps
Wireless & optical node cache size	5Mb,10Mb
Maximum hop distance wireless sensor	3-5m
Maximum hop distance optical sensor	10m
Maximum communication range of the cobot	50m
Topology	Static
Wireless Antenna	Omni-directional
LED (Optical)	Line-of-sight
Path loss exponent for the LoS and non-LoS	1.4, 1.9
The noise floor for the LoS and non-LoS	-89, -97
Shadowing deviation for the LoS and non-LoS	1.12, 1.92
Area: 2D (length×width)	285 × 110m
Simulation time	300 sec
Set of simulations	60

## Ethics Statement

We declare that the manuscript adheres to Ethics in publishing standards and the submitted dataset is the real data recorded in the experiment, and there is no act of stealing other people's data or modifying data.

## CRediT Author Statement

**Muhammad Faheem:** Conceptualization, Methodology, Software, Simulation, Formal analysis, Writing – Original Draft, Project administration; **Rizwan Aslam Butt:** Methodology, Validation, Writing – review & editing.

## Declaration of Competing Interest

The authors declare that they have no known competing financial interests or personal relationships that could have appeared to influence the work reported in this paper.

## Data Availability

Big Dataset of Optical-Wireless Cyber-Physical Systems for Optimizing Manufacturing Services in the Internet of Things-enabled Industry 4.0 (Original data) (Mendeley Data)
